# Exploring the potential of using cattle for malaria vector surveillance and control: a pilot study in western Kenya

**DOI:** 10.1186/s13071-016-1957-8

**Published:** 2017-01-10

**Authors:** Margaret M. Njoroge, Inaki Tirados, Steven W. Lindsay, Glyn A. Vale, Stephen J. Torr, Ulrike Fillinger

**Affiliations:** 1 International Centre of Insect Physiology and Ecology, Thomas Odhiambo Campus, 40305 Mbita, Kenya; 2 Liverpool School of Tropical Medicine, Liverpool, L3 5QA UK; 3 Schools of Biological and Biomedical Sciences, Durham University, Durham, DH1 3LE UK; 4 South African Centre for Epidemiological Modelling and Analysis, University of Stellenbosch, Stellenbosch, South Africa; 5 Warwick Medical School, University of Warwick, Coventry, CV4 7AL UK

**Keywords:** Malaria, *Anopheles*, Vector control, Insecticide-treated cattle, Cattle-baited trap

## Abstract

**Background:**

Malaria vector mosquitoes with exophilic and zoophilic tendencies, or with a high acceptance of alternative blood meal sources when preferred human blood-hosts are unavailable, may help maintain low but constant malaria transmission in areas where indoor vector control has been scaled up. This residual transmission might be addressed by targeting vectors outside the house. Here we investigated the potential of insecticide-treated cattle, as routinely used for control of tsetse and ticks in East Africa, for mosquito control.

**Methods:**

The malaria vector population in the study area was investigated weekly for 8 months using two different trapping tools: light traps indoors and cattle-baited traps (CBTs) outdoors. The effect of the application of the insecticide deltamethrin and the acaricide amitraz on cattle on host-seeking *Anopheles arabiensis* was tested experimentally in field-cages and the impact of deltamethrin-treated cattle explored under field conditions on mosquito densities on household level.

**Results:**

CBTs collected on average 2.8 (95% CI: 1.8–4.2) primary [*Anopheles gambiae* (*s.s.*), *An. arabiensis* and *An. funestus* (*s.s.*)] and 6.3 (95% CI: 3.6–11.3) secondary malaria vectors [*An. ivulorum* and *An. coustani* (*s.l.*)] per trap night and revealed a distinct, complementary seasonality. At the same time on average only 1.4 (95% CI: 0.8–2.3) primary and 1.1 (95% CI: 0.6–2.0) secondary malaria vectors were collected per trap night with light traps indoors. Amitraz had no effect on survival of host-seeking *An. arabiensis* under experimental conditions but deltamethrin increased mosquito mortality (OR 19, 95% CI: 7–50), but only for 1 week. In the field, vector mortality in association with deltamethrin treatment was detected only with CBTs and only immediately after the treatment (OR 0.25, 95% CI: 0.13–0.52).

**Conclusions:**

Entomological sampling with CBTs highlights that targeting cattle for mosquito control has potential since it would not only target naturally zoophilic malaria vectors but also opportunistic feeders that lack access to human hosts as is expected in residual malaria transmission settings. However, the deltamethrin formulation tested here although used widely to treat cattle for tsetse and tick control, is not suitable for the control of malaria vectors since it causes only moderate initial mortality and has little residual activity.

## Background

In sub-Saharan Africa, control of malaria is based largely on the use of long-lasting insecticidal nets (LLINs) and indoor residual spraying (IRS) [1]. These interventions have made a major contribution to malaria control helping to reduce the incidence of clinical disease by 40% between 2000 and 2015 [2]. Applied inside homes, both interventions use insecticides directed at the primary malaria vectors, namely *Anopheles gambiae* (*s.s.*), *An. arabiensis* and *An. funestus* (*s.s.*) that show a strong propensity for entering houses to rest and/or feed [3]. Despite major progress, malaria remains a concern across the continent [1]. Continued residual transmission has been attributed to proportional changes in host-seeking and resting patterns of vectors less affected by indoor interventions [4]. Primary and secondary vectors with naturally more exophilic and zoophilic tendencies or with a higher acceptance of alternative blood meal sources when preferences are unavailable may help maintain low but constant transmission [5–8]. It has been realized that malaria elimination in some areas can be achieved only if residual transmission is addressed adequately. This includes targeting vectors for control outside the house [9–11].

Since *Plasmodium* spp. that cause malaria in humans do not affect livestock, it has previously been proposed that malaria might be controlled by ‘zooprophylaxis’ which diverts zoophilic vectors from humans to livestock [12, 13]. This intervention has been considered for areas where the main malaria vectors are highly zoophilic and exophagic and the livestock population is large. Zooprophylaxis aims to reduce the number of infective bites for humans. However, the evidence collected thus far, has been contradictory and not conclusive on the extent, if any, of the prophylactic effect of animals [12–16].

An alternative method to classical zooprophylaxis would be the direct application of an insecticide on cattle to kill malaria vectors when feeding on the alternative (non-human) host. In principle this approach should be more effective since the insects responding to the cattle would be removed permanently, as against being merely diverted for a while and then remaining free to reproduce and/or subsequently feed on humans. The treatment of cattle with pyrethroids is already an important ‘One Health’ approach for the integrated control of tick- and tsetse-borne pathogens affecting humans and livestock [17]. In East Africa, treatment of cattle with pyrethroids is important for the control of East Coast Fever (ECF), caused by *Theileria parva* transmitted by ticks, and animal African trypanosomiasis, caused by *Trypanosoma vivax* and *T. congolense* transmitted by tsetse. Tsetse flies also transmit *T. brucei rhodesiense* which causes Rhodesian human African trypanosomiasis, a fatal zoonotic disease of humans found in East and southern Africa. Cattle can act as a reservoir host for *T. b. rhodesiense* [18, 19] and the treatment of cattle with pyrethroids [20] is an important component in managing this disease [21], particularly in south-east Uganda [22–24] where most cases of Rhodesian human African trypanosomiasis occur. Malaria is also co-endemic in most of the areas where ECF, animal and human African trypanosomiasis occur. Consequently, the approach might be extended to the control of malaria in areas where malaria vectors feed on cattle. This integrated control of tsetse and mosquitoes has been proposed previously, especially for the Greater Horn region and Maasai steppe of East Africa [25, 26].

The Lake Victoria basin of East Africa is well suited to developing such a ‘One Health’ strategy since it has some of the highest densities of humans and cattle in the region. There is also an increase in zero-grazing practices and consequently an increase in the numbers of cattle close to homes. In some areas of western Kenya, cattle are kept close to houses at night with a large proportion of households actually keeping the livestock in the house where the family sleeps [27]. This provides on the one hand a diverting food source for mosquito populations that feed on animal hosts as well as people [5] and on the other hand, presents an opportunity for killing mosquitoes as they feed on cattle [9].

Topical application of the use of insecticide on cattle to control mosquitoes has been explored in only a few instances [28–30]. A study in Ethiopia [25] proposed that pyrethroid-treated cattle could control malaria where the main vector *An. arabiensis* is largely exophilic and zoophilic. Importantly, the study showed that application of insecticide on cattle did not increase the probability of feeding on humans. Similar findings had been reported from Pakistan for *An. stephensi* and *An. culicifacies* [30].

Here we undertook a study in western Kenya on the shores of Lake Victoria where LLIN ownership and usage is high and vector densities indoors have decreased as a consequence [31–33]. Shifts in the relative abundance of primary vector species have been described [34]; whilst the overall number of *An. gambiae* (*s.l.*) have declined the proportion of *An. arabiensis*, a more exophilic vector, has increased. Furthermore, secondary vectors have been suggested to be playing an increasingly important role in malaria transmission [35]. The objectives of this study were three fold: (i) To determine the knockdown and mortality of *An. arabiensis* feeding on cattle treated with the insecticide deltamethrin or the acaricide amitraz; the latter was included in the study since it was found to be widely used in the study area; (ii) To investigate the abundance and species composition of primary and secondary vectors in the study area with the aim to assess if cattle-targeted interventions could be potentially useful for control of residual malaria. This was done using two methods; indoors with Centers for Disease Control and Prevention (CDC) light traps close to a person and outdoors with cattle-baited traps (CBTs); and (iii) To assess the impact of deltamethrin-treated cattle under field conditions on mosquito densities at the household level.

## Methods

### Study area

Bioassays were conducted at the International Centre of Insect Physiology and Ecology at the Thomas Odhiambo Campus (icipe-TOC) in Mbita, on the shores of Lake Victoria in Homabay County, western Kenya (0°26'06.19"S, 34°12'53.13"E; altitude 1,137 m).

**Fig. 1:**
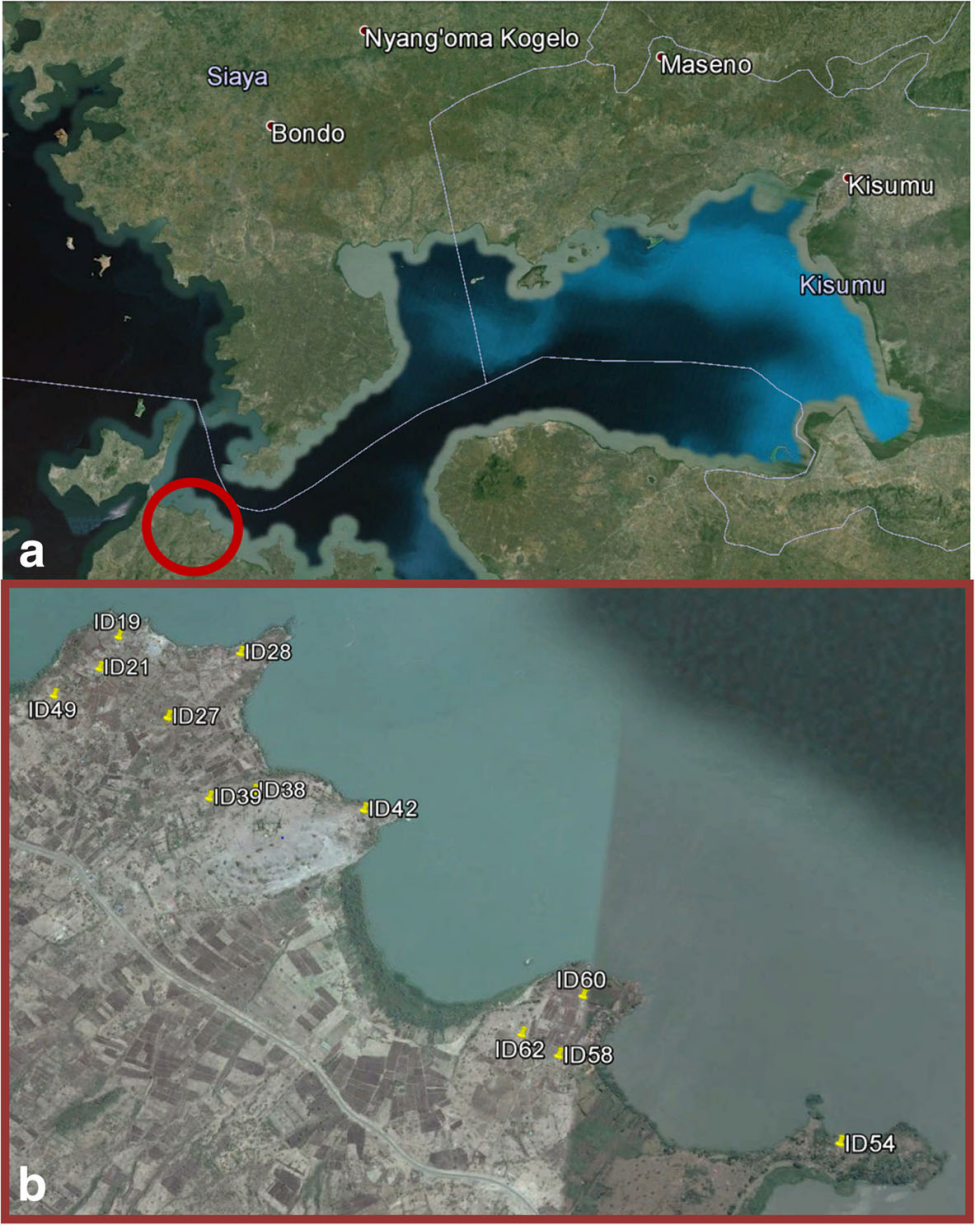
Map of study area and household locations. **a** Overview of Lake Victoria basin area in western Kenya. *Red circle* shows field study area. **b** Field study area showing location of households used for vector sampling and cattle treatment

The field trial was carried out between December 2013 and July 2014 in 12 households in Kirindo (0°26'75.47"S, 34°15'05.48"E) and Kaugege (0°27'37.49"S, 34°16'84.78"E) located 6–8 km from icipe-TOC (Fig. 1). Households were < 500 m from the lake shore consequently in close proximity to aquatic mosquito larval habitats throughout the year. Malaria transmission is perennial and vectors reported for the area include the primary vectors *An. arabiensis*, *An. gambiae* (*s.s.*) and *An. funestus* (*s.s.*), and the secondary vectors *An. rivulorum* and *An. coustani* (*s.l.*). The rainfall pattern in the area is bimodal, with a long wet season occurring between March to June and a shorter and less reliable one between November and December. Rainfall data for the study period were collected from the meteorological station at icipe-TOC.

### Bioassays

#### Mosquitoes

Female *An. arabiensis* Mbita strain were obtained for bioassays from the icipe-TOC mosquito insectary where they were reared following standard procedures [36]. The females were 3–5 day-old and had never fed on blood; they were starved of sugar from noon on the day of their exposure to cattle.

#### Test products and application strategies

For the bioassays, local zebu cattle (males and females, 1–2 year-old, approximately 200–250 kg) were treated with either (i) a 5% w/v emulsifiable concentrate of deltamethrin (Vectocid®, CEVA Santé Animale Africa) diluted with water at 1:1,000, or (ii) a 12.5% w/v emulsifiable concentrate of amitraz (Almatix, Unga Farm Care (EA) Ltd) diluted with water at 1:500 as per manufacturers’ recommendations. The formulations were prepared by adding 2 ml of Vectocid in 2 l of water and 4 ml of Almatix to 2 l of water; this approximates to 28-33 mg/m^2^ deltamethrin and 140–170 mg/m^2^ amitraz when applied equally on the whole body surface area [37]. Two application protocols were tested: (i) restricted application where the full volume was applied to only the underbelly and legs [20]; and (ii) whole body application. A placebo treatment of milky water (2 ml of milk diluted in 2 l of water), simulating the visual appearance of the insecticide preparation, was applied on the control cattle. Applications were made using a high pressure back-pack sprayer. Animals were rented from farms around icipe-TOC with the criterion that they had not received any insecticide, acaricide or endectocide treatments in the immediate 6 months before recruitment. The comparison was repeated for different groups of three animals.

#### Experimental design

**Fig. 2:**
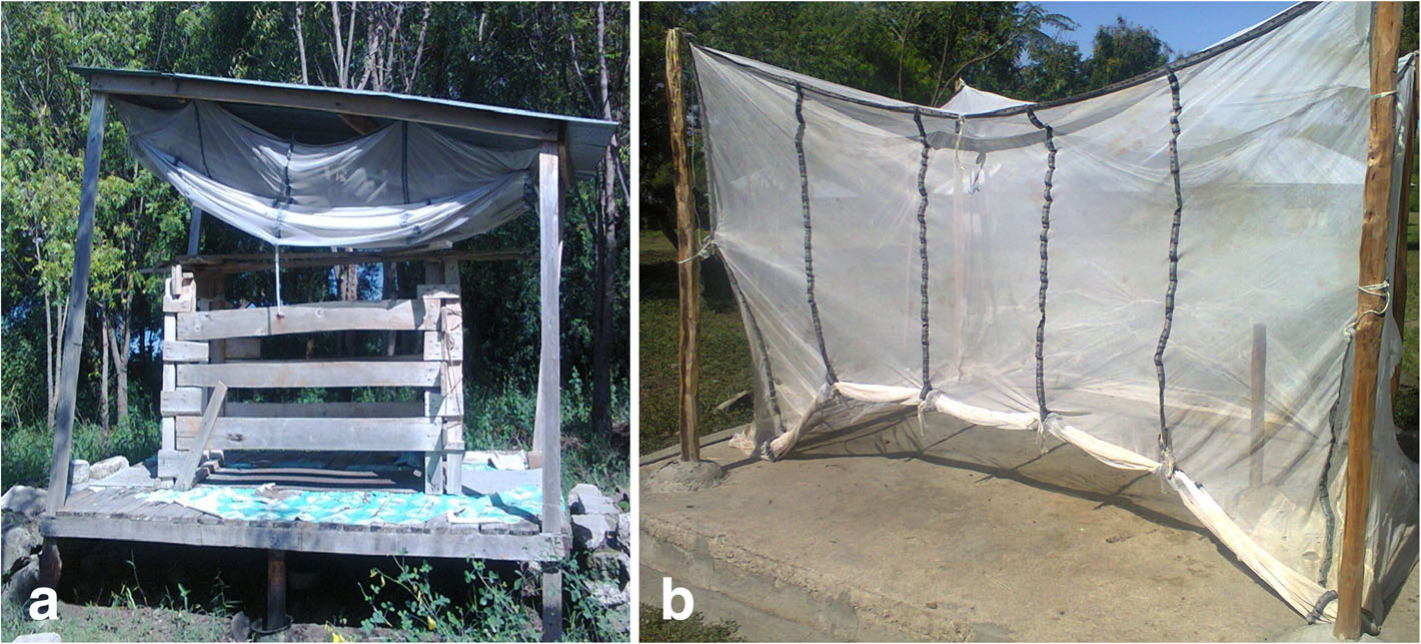
Cattle platforms. **a** Cattle platform and crush for insecticide bioassays with free-flying, host-seeking *Anopheles arabiensis*. **b** Concrete platform covered with netting material used as cattle-baited mosquito trap

Study cattle were placed in retaining crushes mounted on three raised wooden platforms constructed in a secluded area with natural undisturbed vegetation at icipe-TOC. Each platform was 2.5 × 2 m in area and raised 0.5 m above the ground (Fig. 2). Platforms were 20 m apart. To prevent ants from scavenging dead and dying mosquitoes during experiments, each leg of the platform was partially immersed in metal containers filled with water and the rest of the leg was coated with insect-trapping adhesive (Oecotak, Oecos, UK). The platforms were covered with rectangular cotton nets (mesh size 1.2 × 1.2 mm) measuring 2.5 × 2.5 × 2.0 m. The insecticide treatments were compared in a series of replicated Latin squares of 3 platforms × 3 nights × 3 treatments (deltamethrin, amitraz and placebo). The platform, crush and mosquito nets were washed daily to prevent contamination of the cattle. On experimental nights, the cattle were secured inside the platforms at 18:30 h. Comparisons were repeated for 11 groups of three cattle treated with the restricted application protocol. Bioassays were done on the evening of treatment (= day 0) and at 3, 7 and 14 days post-treatment. A second series of experiments was implemented in nine groups of cattle where the whole bodies of the cattle were treated at day 0 and retreated at day 15. Bioassays were implemented on day 0, and thereafter at days 3, 7, 14, 15 (= day 0 of the re-treatment), 18 (3), 25 (7) and 32 (14).

Two types of contact bioassays were implemented in parallel: (i) cup bioassays in which insectary-reared *An. arabiensis* were directly exposed to the treated cattle; and (ii) bioassays in which free-flying mosquitoes released under each net landed and fed naturally on a study animal [25].

#### Cup bioassays

When the animals were placed in the crush, batches of 30 unfed female *An. arabiensis* were exposed in three netting covered cups containing 10 females to the belly of each animal. The cups were kept in position for 3 min and mosquitoes allowed to feed through the netting. After exposure, the mosquitoes were returned to the laboratory and kept under ambient conditions. Knockdown was recorded at 1 h post-exposure and mortality at 24 h.

#### Free-flying mosquito bioassays

After the cup bioassays were done, the nets of the platforms were lowered to enclose the animals. At 19:00 h, 200 unfed female *An. arabiensis* were introduced into the netting enclosure and then collected using mouth aspirators at 22:00 h when they were scored as knocked down or alive. Knocked down and alive mosquitoes were placed separately in 200 ml paper cups which were held in a laboratory at ambient temperature and humidity; the mosquitoes had access to a kitchen paper towel wick soaked in 6% glucose solution. Any mosquitoes missed during the night’s collection were collected at 08:00 h the following morning. All mosquitoes were scored as fed or unfed at time of collection. All mosquitoes collected were scored as dead or alive 24 h after first exposure to the treatment.

#### Confirmation of insecticidal activity of deltamethrin formulation in bioassays with tsetse flies

Previous bioassays of the efficacy of deltamethrin against tsetse have used the Decatix formulation (Cooper Zimbabwe, Harare), a 5% (v/v) suspension concentrate (s.c.) that has been employed routinely in large-scale tsetse control operations in Zimbabwe and elsewhere [20, 38]. The Vectocid formulation of deltamethrin used in the present study had not previously been tested against mosquitoes or tsetse. Given the limited performance of Vectocid for *Anopheles* control in this study, we compared the performance of Vectocid and Decatix against tsetse as an indicator for the insecticidal activity of this formulation.

The comparative studies were carried out at Rekomitjie Research Station (16°7'60"S, 29°24'0"E) in the Zambezi valley of Zimbabwe following a standard method [20, 38] in which wild males and females of *Glossina pallidipes* were caught after they had fed on untreated or treated cattle. Treated cattle were sprayed with Vectocid applied to either (i) the legs and belly only (restricted protocol) or (ii) the whole body at the same concentration used in Kenya. Decatix (5% deltamethrin s.c. diluted at 1 ml/l) was applied to the whole body only.

The fed flies were placed in glass tubes (25 × 75 mm long) which were sealed with netting at one end and a cork at the other. Immediately after feeding the tubes containing the flies were placed in a humidified polystyrene box. At the end of the collection period (14:30–17:30 h), the tubes were transferred to an insectary where they were held at ~25 °C and ~70% RH for 2 h when knockdown was assessed. Comparisons between treated and untreated cattle were carried out for five different groups of animals. Bioassays of tsetse daily continued up to 5 weeks after treatment. The median number of tsetse collected from insecticide-treated cattle per week, pooled across the five comparisons, were 87 (range 55–130) males and 184 (range 117–326) females. For the untreated (i.e. control) animals, the median number of tsetse assayed per week were 31 (9–63) and 75 (24–114) for males and females, respectively, with a total of 529 *G. pallidipes* collected from untreated cattle across the entire trial.

### Field study in western Kenya

#### Household surveys and enrolment

A survey of all households in the study location was carried out to identify those that met the criteria of having at least five cattle tethered close to the houses at night and being > 100 m from other herds and human dwellings. A total of 64 households were mapped and twelve households randomly selected for the trial. Each of the selected households provided informed consent after receiving information about the objectives of the work. The median number of people per household was 13 (range 9–37) and of cattle per household was 14 (range 5–55).

#### Monitoring mosquito populations

Indoor mosquito collections were implemented in all 12 households, in the room where the children (aged 3–14) slept. A standard CDC light trap (John W. Hock, USA) with an incandescent light was suspended 1 m above the floor adjacent to the foot of the bed and operated from 19:00 h until 06:00 h the following morning. Children in the room and all occupants in the house were protected by LLINs (Olyset, Sumitomo Chemical) provided by the study. Cattle-baited trap (CBT) collections were done simultaneously outdoors in the same household. The CBT was constructed within 20–50 m from the house where the CDC trap was placed, at the location where the cattle spent the night. A concrete platform (2 × 2.5 m) was built with a water-filled moat 0.1 m deep and 0.3 m wide to prevent ants from entering and a tethering post was fixed at the centre. A rectangular cotton net (mesh size 1.2 × 1.2 mm), suspended from supporting posts, was draped over the platform (Fig. 2). To collect mosquitoes, an animal from the household was selected by the owner (usually a well-tempered heifer) and was tethered to the post from 18:30 h and the netting material firmly secured on all sides except one where the netting was raised 30 cm above the ground to allow mosquitoes to enter. At 06:00 h the following morning, the raised side of the netting was lowered to enclose all trapped mosquitoes which were then collected using a mouth aspirator. All mosquitoes collected were taken to the laboratory and killed in a freezer. Sampling was done weekly in all households between December 2013 and July 2014.

Mosquitoes were identified morphologically to genus and to species level where possible. Individuals of the *An. gambiae* and *An. funestus* species complexes were identified by polymerase chain reaction (PCR) and gel-electrophoresis [39, 40]. The *An. coustani* group was not further analysed with molecular tools.

#### Study design

Since the bioassays showed that amitraz had no significant effect on the knockdown or mortality of mosquitoes we assessed the impact of deltamethrin-treatment of cattle on mosquito collections in homesteads by comparing herds treated with deltamethrin (test) to herds treated with amitraz (control) as we considered this best practice for animal husbandry. In March 2014, each of the 12 households was allocated to either the deltamethrin or amitraz arms of the trial. Since mosquito densities were highly variable, households were ranked according to their total number of *An. gambiae* (*s.l.*) collected indoors and outdoors during the period November 2013 - March 2014, before the treatments started. Then we randomly allocated one household per consecutive pair of households in the ranked sequence to the deltamethrin arm of the study. Treatment of cattle started on the 15th of April 2014 and ended on 26th of June 2014 with a total of six fortnightly applications applied over 12 weeks. Cattle herds of four households were treated per day and so treatment of all cattle required three days. Within each spraying day, an equal number of herds were treated with amitraz. Between 30 and 70 cattle were treated on each treatment day and the dosage and application was the same as in the experimental bioassays.

#### Susceptibility to deltamethrin

Studies were made of the susceptibility of wild mosquitoes to deltamethrin. Late instar *Anopheles* larvae were collected from aquatic habitats in the study area and brought to icipe-TOC in their habitat water. The larvae were placed in open containers in well-lit netting-screened greenhouses and held under ambient conditions until pupation. They were allowed to grow and develop in water obtained from their wild habitats and food was added sparingly to supplement the nutrients contained in the habitat water.

Pupae were collected and placed in 80 ml emergence cups (7 cm diameter, 4 cm deep). These cups were kept inside 30 × 30 × 30 cm netting-screened mosquito cages and monitored for development and emergence into adult stage. Due to different emergence days, mosquitoes were given a three-day emergence window and then *An. gambiae* (*s.l.*) selected for testing. Mosquitoes were placed in an experimental cage and maintained on 6% glucose solution; humidity was maintained by placing a moist cloth over the cage. When the youngest mosquitoes were three days old, mosquitoes were tested for insecticide resistance following the guidelines of the World Health Organization Pesticide Evaluation Scheme (WHOPES) [41]. In summary, 25 adult female mosquitoes were exposed to deltamethrin insecticide-impregnated papers for one hour and observed for knockdown and mortality up to 24 h. This was replicated three times. Groups of control mosquitoes were exposed to oil-impregnated papers. Molecular species identification of all tested mosquitoes confirmed that all specimens were *An. arabiensis*.

### Statistical analyses

All analyses were carried out using R statistical software [42] or IBM SPSS Statistics 20. Proportions of mosquitoes knocked down or killed in insecticide bioassays were analysed using generalized linear mixed models (*glmer*) fitted with a binomial data distribution and a logit link function generating odd ratios (OR) and their associated confidence intervals (CI). The denominator for the field-cage bioassays was the total number of females recovered per experimental night. The unique animal identifier and the round (cluster of animals treated at the same time) were included in the model as random effects. Treatment type (placebo, amitraz and deltamethrin), day post-treatment and cattle platform identifier were included in the model as fixed factors. The location of the cattle platform had no significant association with the outcome and was removed from the final models. Interaction terms were included for treatment type and day post-treatment. Mean proportions and their associated 95% CI were predicted based on the model parameter estimates. Data from the three cups fixed on the same animal per test day were pooled to provide a single data point.

Results from experimental nights when mortality in the placebo treatment exceeded 20% were excluded from the analysis. Generalized estimating equations were used to analyse the data from the field trial. The trap location (household identification number) was included as repeated measure. Counts were analysed by fitting a negative binomial distribution with log link function. An exchangeable correlation matrix was assumed. Depending on the question to be answered, trapping method (CBT, CDC), months or/and treatment were included as fixed factors in the models. To analyse the impact of the spray week on species counts, the treatment, spray week and the interaction between treatment and spray week were included in the model. All presented means and their 95% CI were modelled as the exponential of the parameter estimates for models with no intercept included.

## Results

### Bioassays

#### Restricted application protocol

**Fig. 3:**
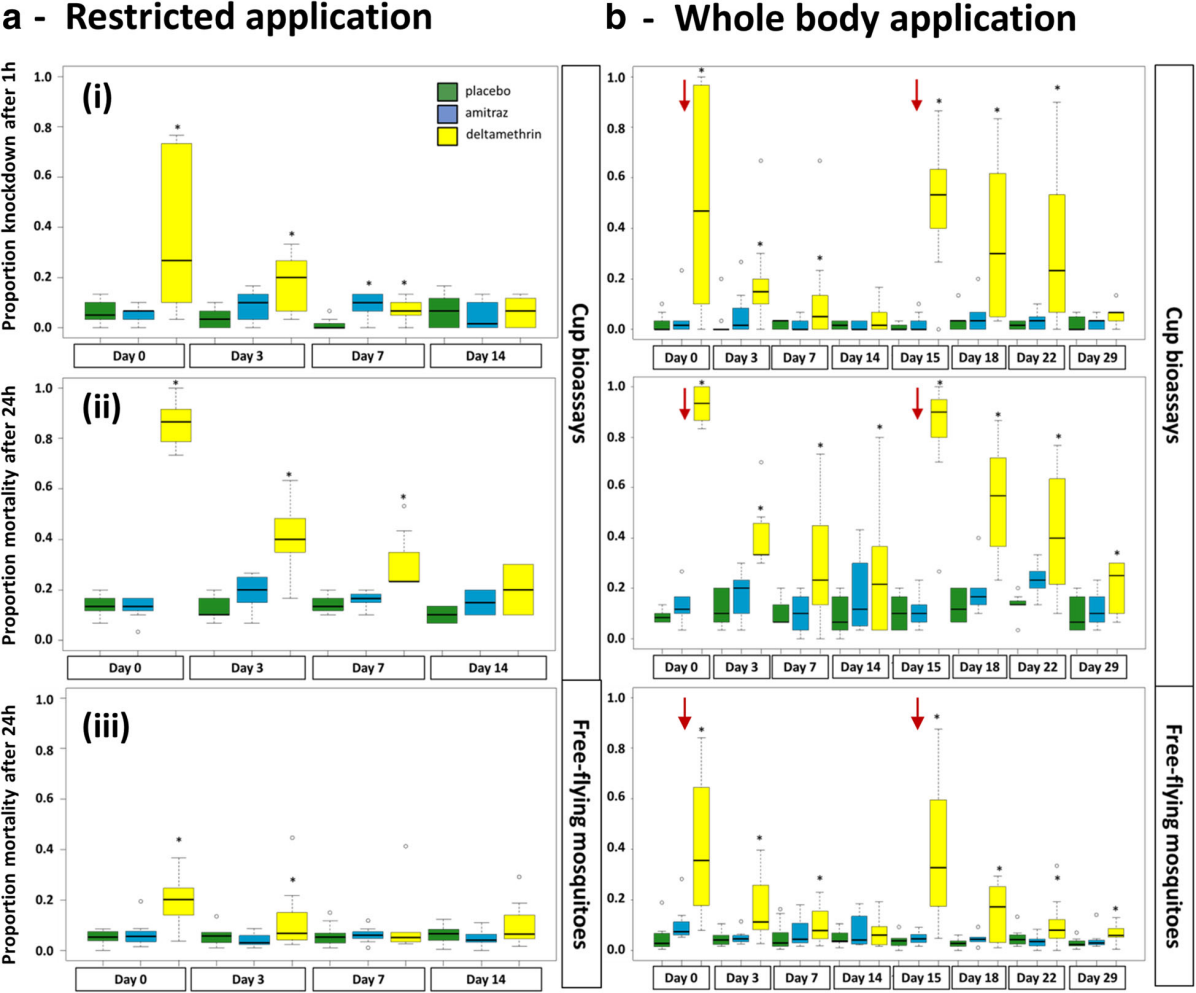
Bioassay results presented as box-plots showing the median proportion of dead *Anopheles arabiensis* exposed to placebo-, amitraz- and deltamethrin-treated cattle. **a** Results from the restricted application protocol. **b** Results from the whole body application protocol; *red* arrows indicate the re-treatment. The graphs in rows (i) and (ii) refer to the cup bioassays (three cups per animal per night, 10 mosquitoes per cup) and in row (iii) to the field-cage bioassays with free-flying mosquitoes (200 mosquitoes per treatment and night). The limits of the boxes indicate the twenty-fifth and seventy-fifth percentiles; the solid line in the box is the median; the capped bars indicate the tenth and the ninetieth percentiles, and data points outside these limits are plotted as circles. Asterisk indicates statistical significance at *P* < 0.05 based on analyses with generalized mixed linear models with animal ID and cluster as random effect and treatment, night and interaction of treatment and night as fixed effect

**Fig. 4:**
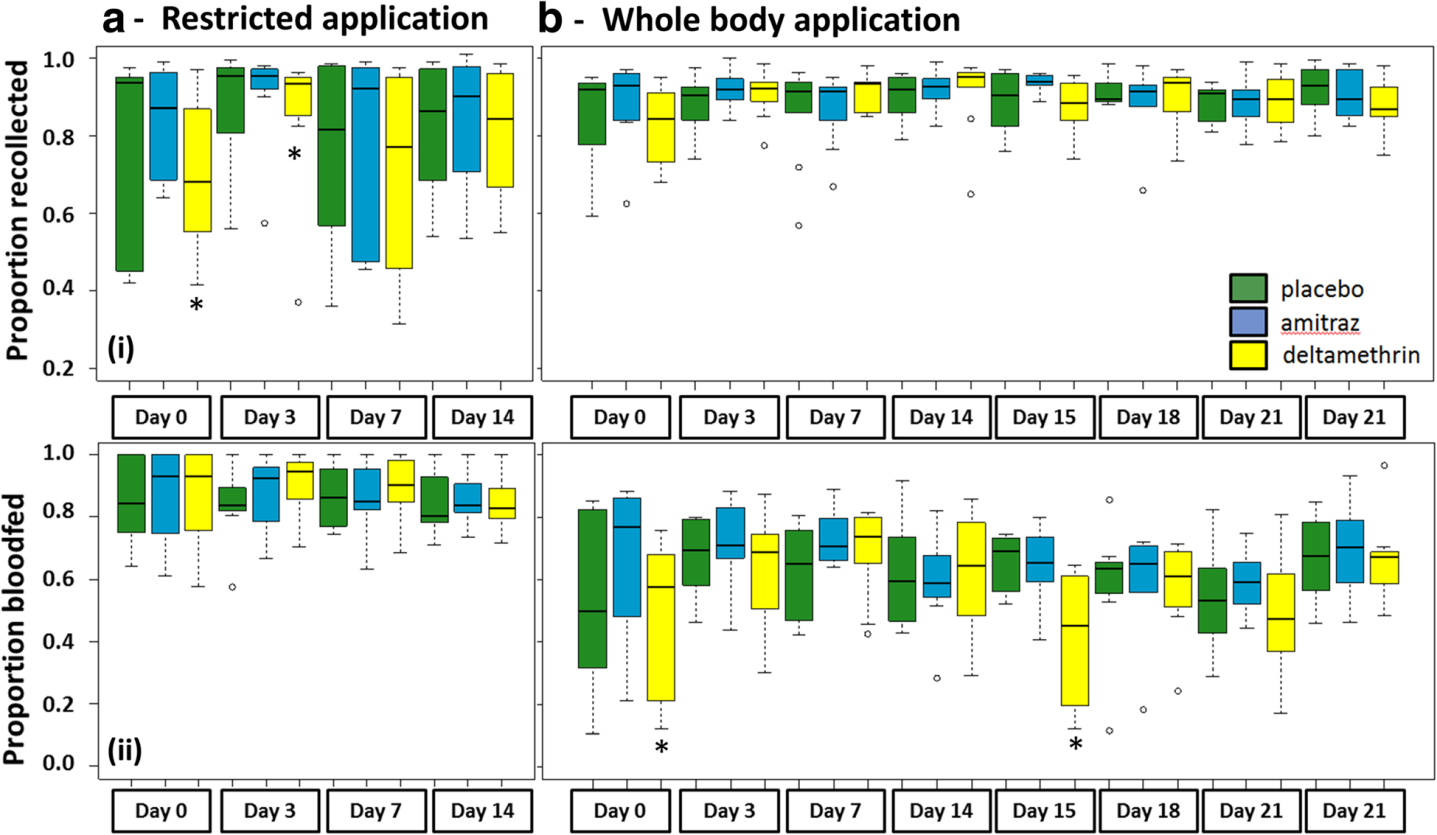
Recollection rates of *Anopheles arabiensis* from field cages. **a** Results from the restricted application protocol. **b** Results from the whole body application protocol. The graphs in row (i) show the rate recollected of all released; the graphs in row (ii) show the rate blood fed of all recollected. The limits of the boxes indicate the twenty-fifth and seventy-fifth percentiles; the *solid line* in the box is the median; the *capped bars* indicate the tenth and the ninetieth percentiles, and data points outside these limits are plotted as *circles*. *Asterisk* indicates statistical significance at *P* < 0.05 based on analyses with generalized mixed linear models with animal ID and cluster as random effect and treatment, night and interaction of treatment and night as fixed effect

Amitraz application on cattle had no significant effect on mosquito survival irrespective of the bioassay method used (Fig. 3), nor on recovery rates (Fig. 4). In contrast, cup bioassays of *An. arabiensis* females exposed to cattle treated with deltamethrin on the underbelly and legs only showed a significant knockdown for up to seven days after application (Fig. 3). Deltamethrin-exposed mosquitoes were 10 times (95% CI: 4–22) more likely to be knocked down than the placebo-exposed (control) mosquitoes for the first week after application. This impact was slightly stronger for mortality recorded 24 h after exposure (Fig. 3). A female mosquito exposed to deltamethrin in cup bioassays on treatment day was 39 times (95% CI: 22–68) more likely to die within 24 h of exposure than a control female. Whilst the natural mortality in the control group remained constant over time, the mean mortality in the deltamethrin group declined quickly. On Day 7 post-application, deltamethrin-exposed mosquitoes were only 2.7 times (95% CI: 1.2–5.9) more likely to die than the placebo group. No significant effect was recorded beyond a week after application.

Under more natural conditions of field cages, the deltamethrin treatment (Fig. 3) was associated with a mortality that was 3.9 (95% CI: 2.1–7.1) times greater than for the placebo group, i.e. still significantly higher than the control but only a tenth of that indicated by the cup bioassays. This treatment effect halved three days post-treatment (interaction between deltamethrin treatment and Day 3: OR 0.5, 95% CI: 0.3–0.8) and was absent on Day 7 and 14 post-treatment. Whilst the impact was significant, the estimated mean mortality rate was only 0.26 (95% CI: 0.16–0.42) on Day 0 and 0.12 (95% CI: 0.07–0.20) on Day 3 compared to 0.05 (95% CI: 0.03–0.09) in the placebo group.

Of all the mosquitoes released in the field cages, a median proportion of 0.9 was recovered, either dead or alive but this varied between nights and treatments, as shown by the interquartile range of 0.63–0.96 (Fig. 4). There was a significantly reduced rate of recovery associated with the deltamethrin treatment on treatment day (i.e. Day 0) and Day 3 post-treatment (OR 0.48, 95% CI: 0.31–0.77) as compared to the recovery rate in the placebo group or to other days.

#### Whole body application protocol

Amitraz applied to the whole body did also not affect mosquito survival in any of the bioassays (Fig. 3). The application of deltamethrin on the whole body improved the impact of the insecticide on host-seeking *An. arabiensis*. As with the restricted application, mortality 24 h after exposure was higher than the 1 h knockdown and there was a significantly higher mortality associated with the deltamethrin treatment up to 14 days after application as compared to the placebo (Fig. 3). A mosquito exposed to deltamethrin in cup bioassays on the day of treatment was 205 times (95% CI: 85–495) more likely to die 24 h after exposure than a mosquito from the placebo group. There was a significant decline in mortality in the deltamethrin group with time. This reduction was more marked for the first treatment interval (Fig. 3). For example, the odds of a mosquito dying in the cup bioassay exposed 3 days after the treatment of the cattle with deltamethrin was 4.7 (95% CI: 1.6–12.3) times greater than the odds of dying in the placebo group in the first round compared to 8.2 (95% CI: 3.1–20.3) times greater in the second round of application. Whilst the impact was still statistically significant 14 days after applications in the cup bioassays, the mean mortality rate was only 0.28 (95% CI: 0.15–0.50) compared to a mortality rate of 0.11 (95% CI: 0.07–0.17) in the placebo group.

The results from the field-cage bioassays with free-flying mosquitoes showed that the overall effect of deltamethrin was less than that with the cup bioassay. Nonetheless, treating the whole body fortnightly produced a larger and longer-lasting effect than when only a restricted application was done (Fig. 3). Mosquitoes exposed to whole body deltamethrin-treated cattle were 19 (95% CI: 7–50) times more likely to die after exposure on application days (Day 0 and Day 15) than those exposed to the placebo-treated cattle. The effect was significantly associated with the test day post-treatment showing a rapid decline. Already 3 days post-treatment this effect was 9-fold reduced (OR 0.11, 95% CI: 0.04–0.40) leading to an estimated mean mortality rate of 0.11 (95% CI: 0.6–0.23). In contrast to the cup bioassay results, no improved residual effect was observed from the field-cage bioassays. Mortality rates were similar on corresponding post-application days during both application rounds (Fig. 3). Overall recovery of released females was better during the whole body application bioassays than during the bioassays with restricted application with a median of 0.91 and an interquartile range of 0.86–0.94 (Fig. 4). Recollection rates were not associated with treatment type or post-treatment day.

#### Impact of insecticide treatment on anopheles blood feeding

While all of the *An. arabiensis* fed when exposed in the cup bioassays, only 0.87 (interquartile range: 0.79–0.96,) of all recovered females had fed in the first series of cage assays when cattle was treated on legs and underbelly only, with no significant effect of treatment types and days post-treatment. For the second series of cage assays, using whole-body treatment of cattle (Fig. 4), 0.65 (interquartile range: 0.52–0.74) fed and there was a significant interaction between feeding and treatment day; females were 1.8–2.8 times less likely to feed on cattle freshly treated with deltamethrin than placebo treated animals (Day 0: OR 0.55, 95% CI: 0.37–0.81; Day 15: OR 0.35, 95% CI: 0.28–0.44). Amitraz had no impact on blood-feeding (Fig. 4).

#### Susceptibility of tsetse

**Fig. 5:**
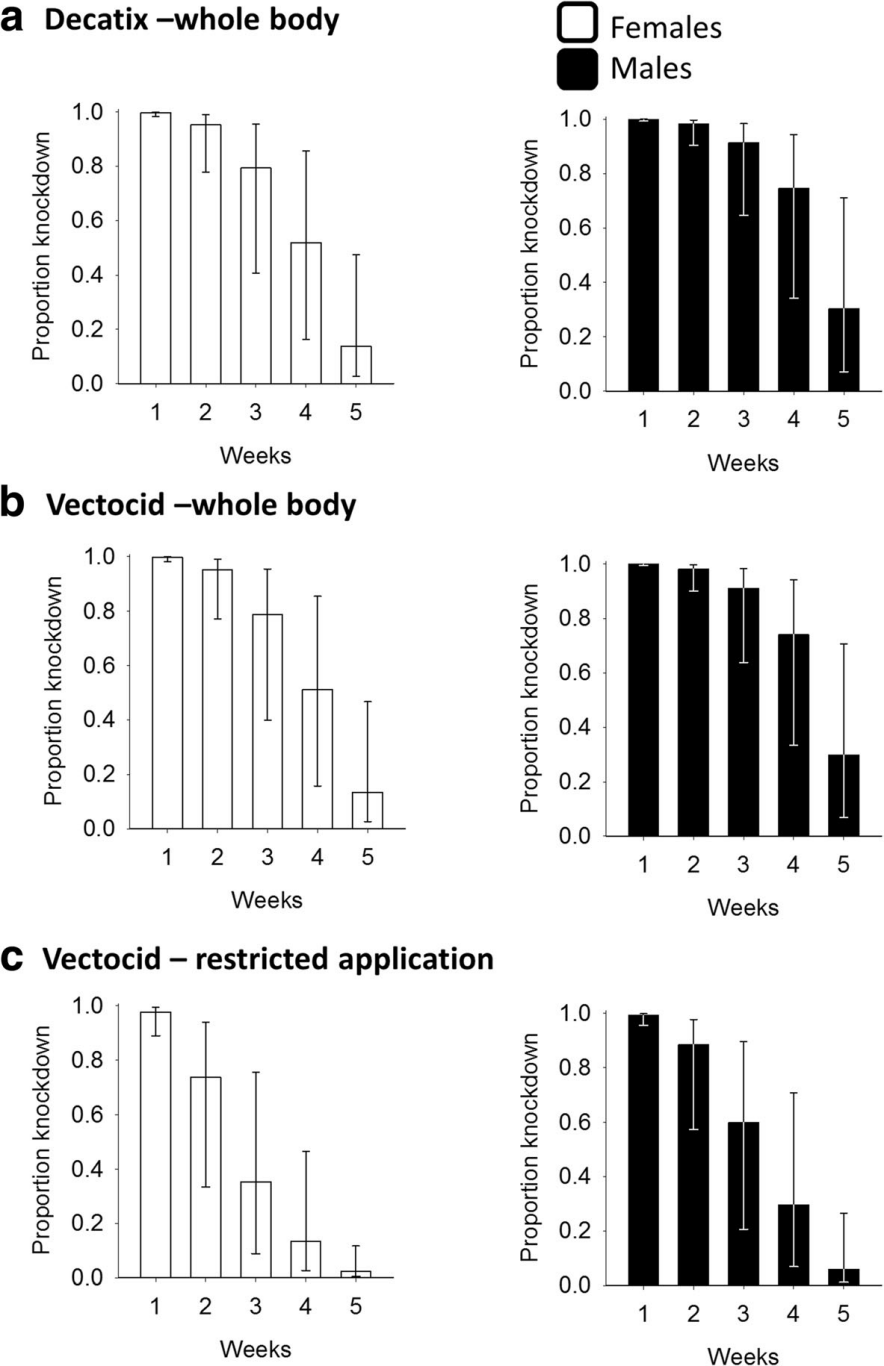
Tsetse knockdown rate in response to two deltamethrin formulations. Proportion knockdown (± 95% CI) of female (open bars) and male (solid bars) *G. pallidipes* exposed to cattle treated with (**a**) Decatix or (**b**) Vectocid applied to the whole body or (**c**) Vectocid applied to the legs and belly only

The percentage knockdown for all tsetse collected from untreated cattle was low (10/529; 1.9%) and so no correction was made for control knockdown in comparisons between insecticide-treated animals. The results (Fig. 5) show that the two deltamethrin formulations applied to the whole body were equally effective, with a knockdown of > 0.5 (i.e. > 50%) for 3 weeks. Restricted application of Vectocid was less effective with the knockdown being > 0.5 for 2 weeks. For both the whole body and restricted applications, the performance of Vectocid against tsetse in Zimbabwe was better than that against *An. arabiensis* in Kenya.

### Field trial

#### Mosquito species composition and population dynamics

**Fig. 6:**
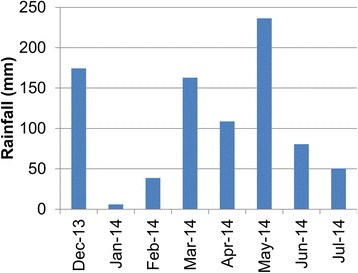
Monthly cumulative rainfall (in mm) in the study area

Over the 8 months a total of 852 mm of rainfall was recorded with increased precipitation during the rainy season March to May (Fig. 6). A total of 14,431 mosquitoes were collected with CBTs, of which 10,942 were culicines (76%) with most (75%) belonging to the genus *Mansonia*. The 3,489 *Anopheles* collected with CBTs belonged to five species: *An. rivulorum* (1,706; 48.9%), *An. arabiensis* (759; 21.8%), *An. coustani* (*s.l.*) (724; 20.8%), *An. funestus* (*s.s.*) (284; 8.1%) and *An. gambiae* (*s.s.*) (16; 0.5%). On average 2.8 (95% CI: 1.8–4.2) primary malaria vectors [*An. gambiae* (*s.s.*), *An. arabiensis* and *An. funestus* (*s.s.*)] and 6.3 (95% CI: 3.6–11.3) secondary malaria vectors (*An. rivulorum* and *An. coustani*) were collected per trap night with CBTs. Only 2% of the *Anopheles* and 4% of the culicines collected in CBTs were male. Of the females, 96% of the *Anopheles* and 93% of the culicines were blood-fed.

Over the same time period, only 2,653 mosquitoes were collected indoors with CDC light traps, of which 1,749 (66%) were culicines, primarily *Mansonia* (75%). Only 904 *Anopheles*, four times fewer than with CBTs, were collected with CDC light traps belonging to the same five species. The species composition was: *An. rivulorum* (379, 41.9%), *An. funestus* (*s.s.*) (328; 36.6%), *An. arabiensis* (155; 17.1%), *An. coustani* (24, 2.7%) and *An. gambiae* (*s.s.*) (18; 2%). On average 1.4 (95% CI: 0.8–2.3) primary malaria vectors and 1.1 (95% CI: 0.6–2.0) secondary malaria vectors were collected per CDC trap night. Males represented 4% of the *Anopheles* catch and 11% of the culicines catch. Of the *Anopheles* females, 22% were blood-fed and of the culicines females 24%.

**Fig. 7:**
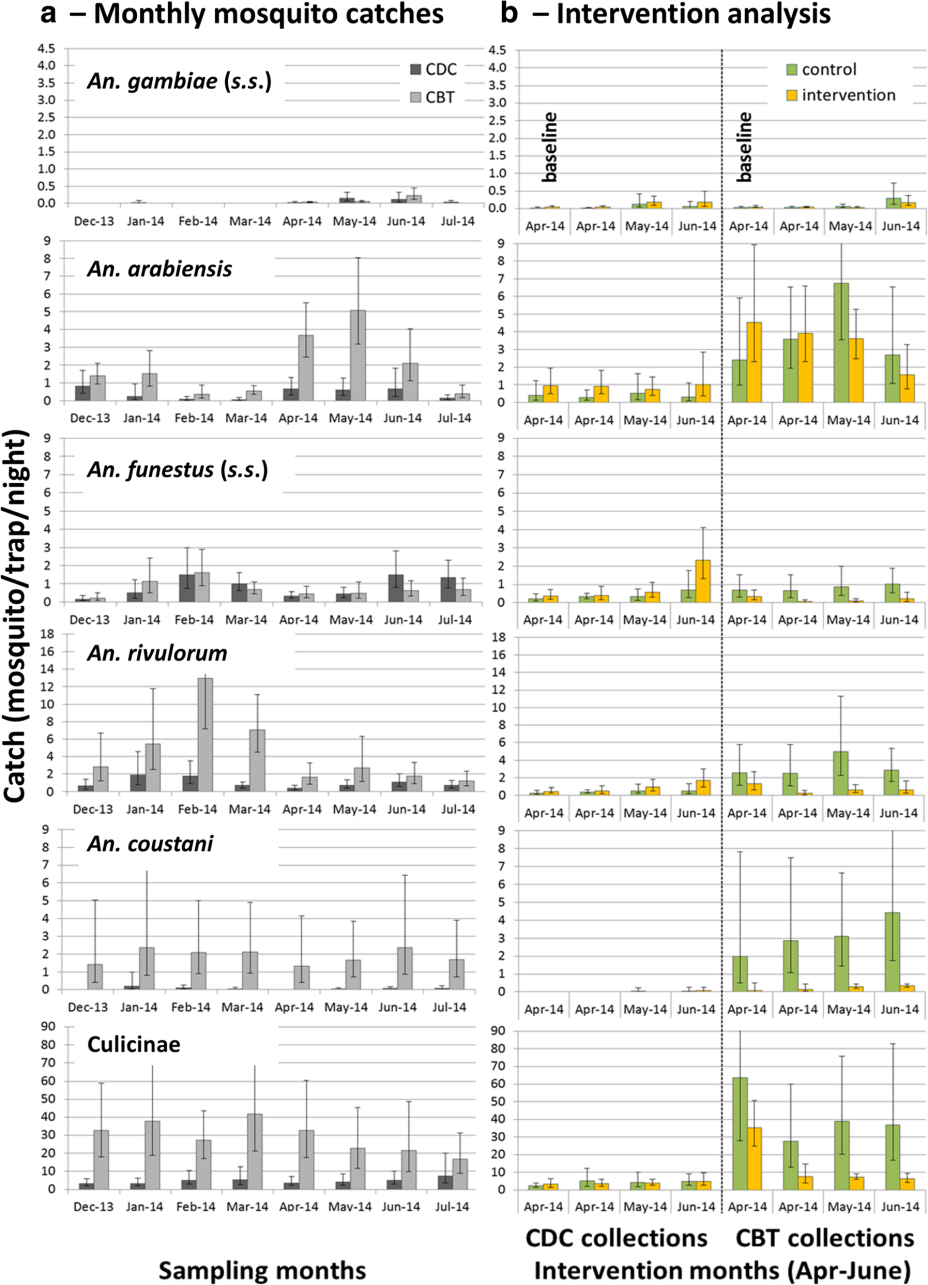
Mean numbers of mosquitoes collected per trap night. **a** Catch per mosquito species per trap night per month for CDC light trap and CBT collections. **b** Catch per mosquito species per trap night for intervention period only, comparing control and intervention sites separately for CDC light traps (left side of graphs) and CBTs (right side of graphs). The first data point from April shows the mean for half the month at baseline. The *vertical bars* show the 95% confidence interval

*Anopheles gambiae* (*s.s.*) was the rarest *Anopheles* species in both indoor and outdoor collections. The probability of collecting a specimen of this species was similar for both collection methods over the 8 months study period (Fig. 7a) with a mean catch per trap night of 0.04 (95% CI: 0.02–0.08). The mean number per trap night of the sibling species *An. arabiensis* was 9 times higher (mean 0.40, 95% CI: 0.23–0.70) in CDC light traps, and 47 times higher (mean 1.98, 95% CI: 1.37–2.86) in CBTs than of *An. gambiae* (*s.s.*). *Anopheles arabiensis* showed a distinct seasonality in the outdoor collections with high numbers at the end of the short rains and a peak during the long rains. This seasonality was not as apparent in the indoor collections (Fig. 7a).

Two sibling species of the *An. funestus* group were identified: *An. funestus* (*s.s.*) and *An. rivulorum*. The mean density of *An. funestus* (*s.s.*) per month was similar when measured with CDC light traps indoors or with CBTs outdoors (Fig. 7a) and was on average per trap night 0.8 (95% CI: 0.5–1.3). *Anopheles rivulorum* was the more abundant species of the complex in both trapping methods and was overall the predominant *Anopheles* species in the study area. *Anopheles rivulorum* was collected in greater numbers in the CBTs. It was 4.5 (95% CI: 3.0–6.9) times more likely to trap an *An. rivulorum* specimen in CBTs (mean per trap night: 4.5, 95% CI: 2.6–7.6) than light traps (mean per trap night: 1.0, 95% CI: 0.6–1.7).

Numbers of the *An. funestus* complex were greatest during the dry season between January and March (Fig. 7a) whereas *An. gambiae* (*s.l.*) peaked in May-June during the wet season. *Anopheles coustani* (*s.l.*) showed no marked seasonality and was almost exclusively collected by the CBTs (Fig. 7a): capture of *An. coustani* (*s.l.*) was 30 (95% CI: 14–66) times more likely outdoors (mean per trap night: 1.9, 95% CI: 0.8–4.5) than indoors. Culicine mosquitoes, representing the largest proportion of mosquitoes collected with either method, were collected in similar numbers throughout the study (Fig. 7a). However, the probability of collecting a specimen with CBTs was seven times higher (95% CI: 4.7–9.9) than with light traps.

#### Impact of insecticide treatment on mosquitoes

There was no significant effect of the intervention (deltamethrin vs. amitraz) on the mean monthly mosquito numbers collected by the two types of traps (Fig. 7b). Differences between intervention and control densities for *An. funestus* (*s.s.*), *An. rivulorum* and *An. coustani* (*s.l.*) in the CBTs (Fig. 7b) need to be interpreted with caution since these differences existed before any cattle were sprayed, as shown in Fig. 7b for the first half of April.

**Fig. 8:**
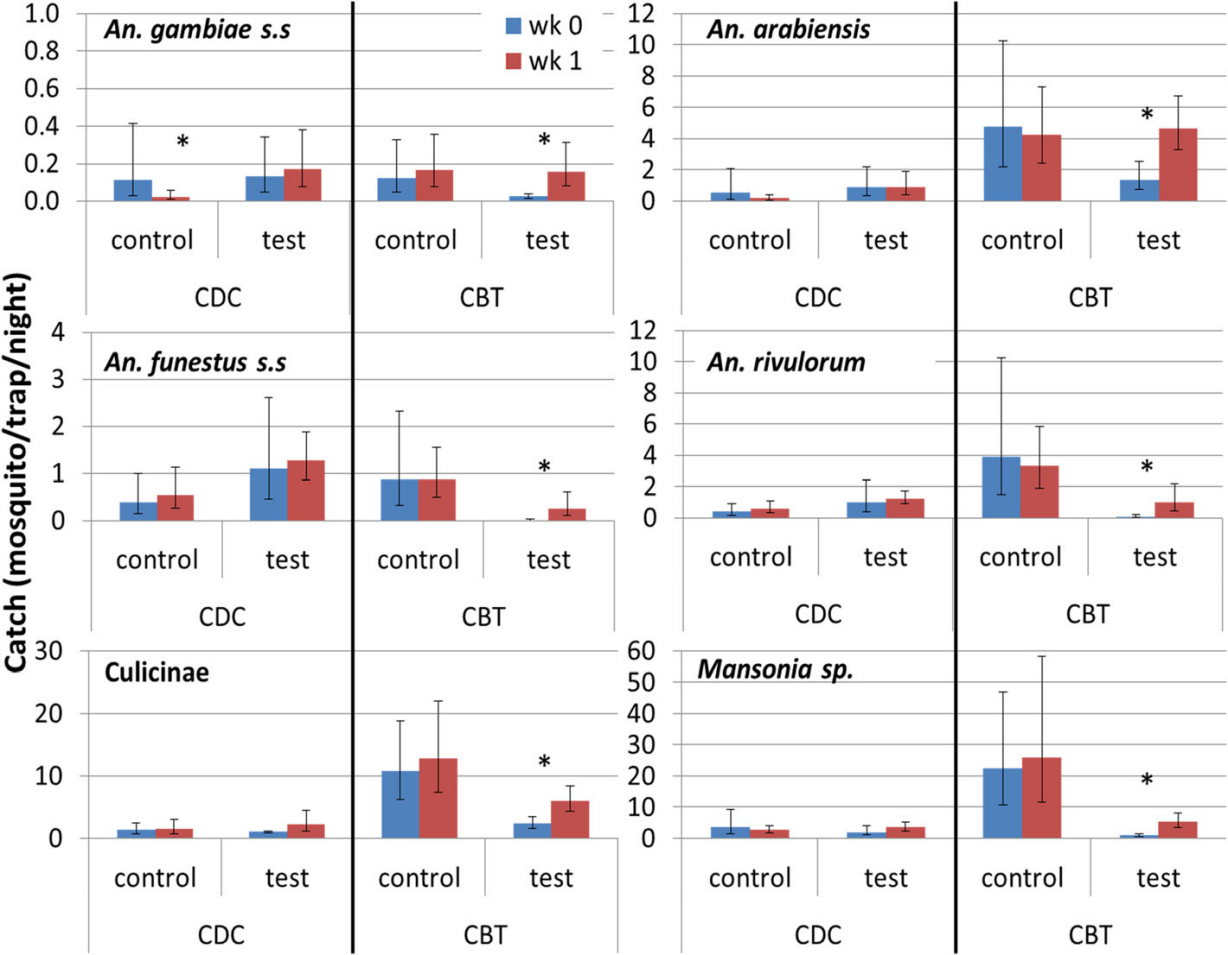
Comparison of deltamethrin impact in field immediately after treatment (wk. 0) and 1 week later (wk. 1). Mean mosquito catch per trap night in intervention period separate for CDC light trap collections and cattle-baited trap (CBT) collections in control and intervention (test) sites. *Anopheles coustani* was not included in this analysis due to high baseline variability. The *vertical bars* show the 95% confidence interval. *Asterisk* indicates statistical significance at *P* < 0.05 based on analyses with generalized mixed linear models

Results from the bioassays showed a significant effect for the week following treatment and we therefore analysed the data to determine whether a similar transient effect occurred in the field study. The results (Fig. 8) show that while there was no significant impact on mosquitoes caught indoors, there was indeed a significant reduction in the number of mosquitoes caught on cattle immediately after treatment compared to the following week (8 days post-treatment). The mean number of mosquitoes collected per CBT night was similar in both weeks and for all species for the amitraz-treated herds but was significantly lower immediately after treatment than 1 week later for all species in the deltamethrin-treated herds (Fig. 8). On average for all *Anopheles* species combined it was 4 times less likely (OR 0.25, 95% CI: 0.13–0.52) to catch a live specimen with CBTs immediately after treatment than 1 week later.

#### Susceptibility of wild An. arabiensis to deltamethrin

The mean mortality of wild *An. arabiensis* exposed to deltamethrin-treated papers was 0.89 (95% CI: 0.81–0.94) compared to the control of 0.18 (95% CI: 0.09–0.31). Since the total mortality in the control group was higher that 15%, Abbots correction [43] was performed resulting in a corrected mortality of 0.87 (95% CI: 0.80–0.93).

## Discussion

Our results show that the current practices and insecticide/acaricide formulations used to control tsetse and ticks in East Africa are unlikely to have a marked effect on malaria vector mosquitoes. The bioassays revealed that amitraz, an amidine acaricide widely used to control ticks [24] and previously described to have repellent activity [44], has neither repelling nor killing effects against *Anopheles* mosquitoes, whilst the deltamethrin formulation at the concentration and application rates recommended against ticks and tsetse had a moderate (<50% mortality) and short-lived (~1 week) impact on survival in experiments with free-flying mosquitoes in large field cages and in the field. We confirm observations from other studies [25] that cup bioassays largely overestimate the actual impact of the treatment on mosquito survival emphasizing the need for testing of insecticides under as natural conditions as possible. The performance observed here of deltamethrin was comparable to that reported from similar studies conducted in Ethiopia [25]. In contrast, a study of malaria mosquitoes exposed to deltamethrin-treated cattle in Pakistan found mortality persisting at around 40% for up to 2 weeks [28]. Potential explanations for this difference include the formulation used, application of insecticide using sponges rather than spraying and differences in the susceptibility of the mosquito populations. The restricted application of deltamethrin to the underbelly and legs only, as practiced for tsetse control to reduce costs and environmental impact [20] and as suggested for mosquito control [25], did not show the expected results. Whole body application increased the mortality rate in our experiments significantly.

Results from the field trial confirmed that treating cattle with deltamethrin does not produce a marked reduction in the numbers of malaria mosquitoes. However, the absence of any measurable impact on monthly mean mosquito numbers is not surprising given the rapid decline of the killing effect of deltamethrin within a week as shown in the bioassays. Moreover, the small number of herds treated in this pilot study would not lead to a community-wide reduction in mosquito populations. Consistent with the results from the bioassays, there was a significant decline in the numbers of mosquitoes caught in CBTs immediately after the treatment of the animals but was not detectable after seven days. This reduction was likely due to the combination of immediate toxic and possibly repellent effects. Mosquitoes that entered the trap, fed and died during the night were less easy to find the following morning, thereby reducing the number of mosquitoes recovered. In addition we recorded in the field-cage bioassays a reduced proportion of females that were blood-fed on treatment days suggesting a repellent effect of the treatment. However, studies of *An. arabiensis* in Ethiopia found that treating cattle with deltamethrin did not reduce the numbers of mosquitoes landing on them but it did reduce the proportion that fed [25] suggesting that the repellent effect is not long-range.

A greater impact on mosquito survival might be expected if the intervention was applied with higher frequency (e.g. weekly). However, fortnightly application is the highest application rate currently permitted for these products. In practice, resource-poor livestock keepers seldom treat their cattle so frequently. A recent mixed-methods study of 495 livestock keepers in Uganda suggested that 37% treated their cattle at fortnightly intervals during the wet season [24]. Moreover, programmes to control Rhodesian sleeping sickness aim to treat ~20% of cattle each month [21, 22, 24]. Hence it seems unlikely that farmers treating their cattle primarily to control ticks and tsetse would achieve application rates higher than those tested in this study. Furthermore, the insecticide tested is identical to those used for mosquito control indoors and subject to increasing levels of resistance [45]. Accordingly, treating cattle for mosquito control with pyrethroids is unlikely to be sustainable and frequent application would contribute to even more rapid development of resistance [46]. Already, deltamethrin resistance of *An. arabiensis* from the study area was documented with the WHO tube bioassays, where mortality was less than 90%. Recent publications also report growing pyrethroid resistance in a number of the malaria vector species in the study area [35, 47, 48]. This might have also contributed to the limited effect of the field intervention.

Studying the *Anopheles* population with two different trapping tools in parallel provided important insights into species composition and abundance. We found five *Anopheles* species that have the potential of transmitting malaria occurring in relatively small numbers but throughout the year. Ten to 15 years ago, in the same study area, CDC light traps operated indoors caught relatively large numbers (> 30 per trap night) of *Anopheles*, comprising roughly equal proportions of *An. gambiae* (*s.s.*) and *An. arabiensis* [49]. In the present study, the abundance of *An. gambiae* (*s.s.*) was extremely low; we caught a total of 34 over a period of 7 months or 762 trap nights. Notably, these were collected in similar numbers with CDC light traps around human hosts and with CBTs. *Anopheles arabiensis* however were 9 times more abundant in light traps and 47 times more abundant in CBT than *An. gambiae* (*s.s.*) confirming a species shift in recent years within the *An. gambiae* complex likely due to indoor vector control as described for other areas [50, 51]. Similarly, *An. rivulorum*, the more exophilic member of the *An. funestus* group dominated the catches in both trapping methods over *An. funestus* (*s.s.*), and was in fact the most abundant *Anopheles* species collected. It has been suggested recently for the study area that *An. rivulorum* is involved in malaria transmission with a sporozoite rate similar to that of *An. funestus* (*s.s.*) [35] and historical literature highlights the potential of this vector to maintain malaria transmission after the control or elimination of the major endophilic vectors [52]. The mosquitoes collected with CBTs show clearly the high abundance of this species in the study area which underlines the need to investigate the role of so-called secondary vectors in residual malaria transmission and the need for additional vector control tools targeting exophilic species. Another potential malaria vector in the study area was *An. coustani* (*s.l.*). This species would have been nearly overlooked if only indoor collections had been implemented, since few enter houses. Sporozoite infection rates have not yet been investigated for this species in the study area and testing was beyond the scope of this project. However, an increasing number of reports suggest an important role for *An. coustani* as a malaria vector in changing transmission settings in East Africa [51] and beyond [53, 54]. Due to its absence from the indoor environment [55], this vector will not be adequately targeted by current vector control interventions.

It needs to be emphasized that the aim of using the two trapping tools was not to compare biting rates directly but rather to characterise the vector population as it presents itself with the two tools. Clearly, we do not know how mosquitoes collected from CBTs relate to outdoor biting on humans in this particular setting. Also the trap type compounds the catch size and the trapping efficiency of light traps and CBTs certainly differs. Here our aim was to gauge if the standard method of monitoring adult vectors with CDC light traps close to a protected human volunteer indoors does represent the potential malaria vector species composition, overall abundance and seasonality in an area where LLIN coverage is high. The CBT collections suggest that far higher numbers of vectors are present in the study area than that suggested by the CDC light trap collections. Whilst the same five vector species were collected with both trapping methods, over five times more *An. arabiensis* and secondary vectors were recorded with the CBTs and *An. gambiae* (*s.s.*) and *An. funestus* (*s.s.*), generally considered highly anthropophilic, were notably recorded outdoors on cattle in a similar density as indoors close to humans. Of notable interest was the readiness of *An. funestus* (*s.s.*) to feed on cattle which was in line with what had recently been reported from Tanzania [56]. This supports the hypothesis that cattle-targeted vector control interventions would not only target naturally zoophilic vectors but would also affect opportunistic feeders that lack access to human hosts as is expected in residual transmission settings.

The CBT collections also revealed a distinct seasonality for different vector species, with *An. coustani* being present throughout the year, whilst the *An. funestus* group showed higher densities during the drier months and the *An. gambiae* group during the rainy season. Potentially, this could indicate that different vectors are responsible for malaria transmission throughout the year. Further research is required to investigate this. Whether all vectors collected on cattle may also have fed on humans remains uncertain. However, the presence of all species indoors and the recent report of sporozoite infections in *An. rivulorum*, suggest that humans are amongst the blood hosts.

There is a pressing need to reassess methods of monitoring mosquito vectors. Human-landing catches performed outdoors would sample exophagic species but concerns about the ethics and safety means that this method is seldom used. Cattle-baited traps would be effective and might be more frequently considered in research programmes since we show that they not only attract vectors known to be highly or partly zoophilic but also represent the primary, anthropophilic vectors similarly well as human-baited light trap collections indoors. While the CBTs used here are not convenient for routine mosquito surveillance in large-scale vector control programmes, they do suggest that there is an opportunity to develop additional tools for sampling mosquitoes outdoors. The ubiquity of cattle in rural areas of sub-Saharan Africa provides a natural and abundant source of host odours which could be exploited for sampling systems. The much larger sample of malaria and other mosquito-borne disease vectors, i.e. *Mansonia* vectors of lymphatic filariasis, from CBTs also provides a better opportunity for screening a large number of samples for sporozoite infections and infections of bacteria and viruses that are usually rare.

## Conclusions

The deltamethrin formulation used to treat cattle for tsetse and tick control tested here is not suitable for the control of malaria vectors since it causes only moderate initial mortality and has no residual activity. Research into long-lasting formulations persisting at least for 1 month would be desirable. However, given that pyrethroids are the only class of insecticide currently licensed to treat mosquito nets, and the increasing resistance of malaria vectors against pyrethroids insecticides [45], it would be wise to investigate alternatives with a completely different mode of action [9, 46]. Our entomological sampling with CBTs highlights that targeting cattle for mosquito control has potential. Cattle-targeted interventions would not only target zoophilic malaria vectors but also affect opportunistic feeders that feed on animals when human hosts are less available. Such a situation is likely in residual malaria transmission settings where a large proportion of the human population use insecticidal bed nets. Cattle-based intervention would also target other genera of mosquito vectors responsible for human and animal diseases [57, 58]. Cattle-targeted interventions offer an opportunity to develop ‘One Health’ integrated vector management tools aimed at improving human and animal health. Such an approach would be attractive to farmers and might ensure better adherence to treatment regimens [59].

## Abbreviations

CBT: Cattle-baited traps
CDC: Centers for disease control and prevention
ECF: East coast fever
icipe-TOC: International centre of insect physiology and ecology - Thomas Odhiambo campus
IRS: Indoor residual spraying
LLIN: Long-lasting insecticidal nets
WHO: World Health Organization
